# The Impact of Educational Intervention Based on Empowerment Model in Preventing Violence Against Women

**DOI:** 10.5812/ircmj.14432

**Published:** 2014-07-05

**Authors:** Mohammad Hossein Taghdisi, Fatemeh Estebsari, Maryam Dastoorpour, Ensiyeh Jamshidi, Fiesal Jamalzadeh, Marzieh Latifi

**Affiliations:** 1 Department of Education and Promotion, School of Public Health, Iran University of Medical Sciences, Tehran, IR Iran; 2 Community Based Participatory Research Center, Iranian Institute for Reduction of High-Risk Behaviors, Tehran University of Medical Sciences, Tehran, IR Iran; 3 Research Center for Modeling in Health, Institute for Futures Studies in Health, Kerman University of Medical Sciences, Kerman, IR Iran; 4 Department of Health Economics, Kerman University of Medical Sciences, Kerman, IR Iran; 5 Department of Health Education and Promotion, School of Public Health, Shahid Beheshti University of Medical Sciences, Tehran, IR Iran

**Keywords:** Violence, Self Esteem, Self Efficacy, Empowerment, Women

## Abstract

**Background:**

One of the most obvious forms of violence in today's society is violence against women. In Iran, along with other countries, violence against women has become a problematic issue.

**Objectives:**

The present research aims to investigate the impact of educational intervention based on empowerment model in preventing violent behaviors against women.

**Patients and Methods:**

The present study is an intervention research done through the random selection of 91 women under the aegis of Imam Khomeini Relief Foundation in Gorgan. Tools for data gathering included demographics checklist, Rosenberg Self-Esteem, general self-efficacy, awareness and attitude questionnaires. Three ninety-minute educational sessions were held for each group to enhance their awareness, change their attitudes, and train them life skills to increase self-esteem so that they can express their vicarious experiences to increase their self-efficacy toward violent behavior. Following the post-test, data were analyzed with SPSS software (version 20). Tests for analyzing data included descriptive and analytical tests (chi-square, Pearson's correlation, independent samples t-test, One-way ANOVA and paired t test).

**Results:**

Results indicated that the frequency of domestic violence against participating women was significant after educational intervention, as compared to pre-intervention period. Paired t-test showed that average scores of awareness, attitude, self-esteem, and self-efficacy constructs, and total power were statistically higher after educational intervention as compared to the period prior to intervention.

**Conclusions:**

As one of the manifestations and the moving force of empowerment, education is the first major strategy in codifying, designing, and implementing empowerment programs. For women to be empowered, the active participation of all people in education is required.

## 1. Background

Violence has always been one of humanity’s experiences. Its effects can be seen around the world. World Health Organization (WHO) defined violence as the intentional use of physical force or strength, with threat or desire, against another or self, group or society, which results in physical or mental injuries, death, growth deficiencies or various types of deprivation. In Iran - like all over the world - women are prone to domestic abuse and violence for different reasons, including inferior physical strength. In a study of Balali Meibodi, the prevalence of women violence in Kerman was 46%. In Eastern India, prevalence of physical violence, psychological violence, and sexual violence were 16%, 52% and 25%, respectively. The prevalence of the overall life intimate partner in Erbil City of the Kurdistan region of Iraq was 58.6%. In western of Ethiopia during past 12 months, the prevalence of intimate partner violence against women was 76.5% (1). One key strategy in promoting health is to strengthen people in society; this means empowering societies and increasing their ownership and control over their own fate. The objective of empowerment in health promotion is to empower individuals and societies so that they may make the best decisions in the field of health, considering the importance of health determinants, which are of great importance in the physical and social atmosphere (2). Empowerment and health promotion decrease inequalities and spread justice (3). It implies the promotion of a positive sense of trust, adaptability and power control and helps others reach their goals (4). The basics of empowerment include the problem-solving ability, self-dependency and self-confidence creativity. Empowerment is a fundamental part of health promotion in society. Nowadays, women make up half of society’s population and play a vital role in building the society. Therefore, paying careful attention to their empowerment can lead to a stable society development (5). For a family to function well, peace and security have to settle in its core. Minor family disputes are associated with the actions and structural base of the family, whereas those on a larger scale are associated with social structures. A family becomes problematic when it cannot realize its goals, especially when it stays far from its main objective i.e. being a primary social group (6). In a global population study, 10% to 69% of the women reported that at some point in their lives they had been sexually abused by either their husband or another male relative (7). Women’s abuse in the family can have a broad range of social consequences. The effects of abuse on children can be both direct (the aftereffects of a couple's quarrel observed by the child), and indirect (a mother mentally unloading herself by beating her child), resulting in outcomes such as the children’s mistreatment, neglect or even abuse (8). In order to help families reach a goal in health care, the methodology must be based on their empowerment, which includes positive self-confidence, goal reaching ability, having a sense of control over life and changing processes and also hopeful feeling (9). Empowerment is a fundamental part of health promotion in society, and its basics include problem-solving, self-dependence and self-confidence (10). Empowerment means helping the family so that it can reach the ability to change (11). The feelings of participation and belonging to the society are vital and inter-dependent. During the empowerment process, women become aware of their personal needs and demands. They find the courage to reach their goals and become capable in actualizing their demands (1). Teaching women about self-esteem, the benefits of empowerment, sexual awareness, gaining certainty and worthiness, actualizing appropriate knowledge and skills and finally higher levels of education are some of the actions that may lead to women’s empowerment. Being completely aware of one’s rights is an empowerment tool that has been used by women and even men (12).

## 2. Objectives

Previous studies indicated high frequency of abuse in families and its consequences. Considering the importance of interventions such as educational interventions (inside a theoretic framework), in order to increase women’s abilities to prevent abusive behaviors, the current research aims to determine the impact of an empowerment program on prevention of abusive behavior toward women and propose solutions for less damages and consequences.

## 3. Patients and Methods

### 3.1. Study Population and Sampling

Once the approval was gained from Ethics Committee of Tehran University of Medical Sciences (grant number: 16418 and IRCT registration) and required arrangements were done, this quasi-experimental study with pretest and posttest design was conducted on 91 married women under the aegis of Imam Khomeini Relief Foundation in Gorgan in Winter 1390 and Spring 1391. In this research, according to Cochran formula with 36% violence against women, 12% error limit, 80% test power, and 95% certainty level, a sample size of 126 individuals were estimated. Given the 15% loss for various reasons (such as the possibility of leaving the study during intervention (10%), and possibility of leaving the study during follow-up (5%)) and given the limited population of the research, 91 individuals were determined eventually and these individuals were selected with simple random sampling from the list of women. Those who entered the study needed to 1. Be married, 2. Be under the aegis of Relief Foundation at least for a year, 3. Have at least one child, 4. Be originally from Iran. In addition, the criteria for leaving the study were: 1. not participating in two educational sessions, 2. Immigrating from Gorgan, and 3. not being interested in continuing the cooperation.

### 3.2. Data Collection

Data collection instruments included:

1. Demographics checklist including age, education (illiterate, elementary, junior high school, high school, and more), length of married life, number of children, children gender (son, daughter, son and daughter), living with husband's family (yes, no), employment status (housewife, employed), house ownership (tenant or landlord), living place (city, village), husband's education (illiterate, elementary, junior high school, high school, and more), husband's employment status (employee, self-employed, jobless), husband's smoking (yes, no), husband's addiction (yes, no), domestic violence against participating women in the last 12 months (yes, no) (before and after educational intervention).
2. Researcher's questionnaire based on empowerment constructs, which consisted of four sections:
  - Researcher's built questionnaire about women awareness of domestic violence: This tool includes 11 questions based on a Likert scale "yes, no, and I don't know" in which "yes" answers got one point and "no" and "I don't know" answers got zero point. The total score of the individual determined her level of awareness.
  - Researcher's built questionnaire concerning women attitude toward domestic violence: This tool includes 10 questions on a Likert scale "agree", "disagree", and "no idea". To evaluate the attitude of individuals under study, questions 1, 2, and 10 in this questionnaire got one point for "disagree" answers and zero point for "agree" and "no idea" answers. Also, questions 3, 4, 5, 6, 7, 8, and 9 got one point for "agree" and zero point for "disagree" and "no idea" answers. Finally, the total score of the individual determined her attitude about domestic violence. It is noteworthy to mention that the content validity of women awareness and their attitude concerning domestic violence were evaluated in this study first by the confirmation of 10 faculty members of health faculty based on the latest professional texts and reliability of these tools were confirmed by test-retest method. In this method, awareness and attitude questionnaires were given twice, with a 14-day interval in between to 10 individuals who were qualified to enter the study. Their responses for every question were investigated twice regarding their continuity and homogeneity and correlation coefficients 0.88 and 0.81 were achieved for awareness and women attitude toward domestic violence questionnaires respectively.
  - Rosenberg Self-Esteem Questionnaire: This tool was designed by Maurice Rosenberg in 1965 (13). This questionnaire includes 10 questions on a Likert scale "strongly agree", "agree", "disagree", and "strongly disagree", in which "strongly agree" and "agree" answers got one point and "disagree" and "strongly disagree" got no point. Finally, the total score of individuals determined women self-esteem regarding domestic violence. The questionnaire of Bohloo and Rajabi's study which was conducted on 129 students of Shahid Chamran University of Ahvaz confirmed the validity and reliability of this tool (14) and thus it was suitable for this study as well.
  - Jerusalem and Schwarzer Standard Questionnaire of Perceived General Self-Efficacy (15): This questionnaire includes 10 questions on a Likert scale "exactly true", "moderately true", "hardly true", and "not at all true" in which "exactly true" and "moderately true" answers got one point and "hardly true" and "not at all true" answers got no point. Eventually, the total score of individuals determined self-efficacy of participating women. The Persian version of this tool was used in this research (2).

### 3.3. Intervention Program

Questionnaires were filled out in the first stage (pre-test) of the research. Then, given the results of pre-test, educational planning was done and appropriate educational packages were prepared and implemented for individuals under study. Type, content, and method of education, as well as the number and appropriate time for educational classes were determined based on the analysis of results on pre-test stage and required educational materials were designed. Based on the results of analysis at this stage, educational intervention was designed according to self-efficacy theory in three 60-minute sessions using the method of group discussion, question and answer, consulting, practical display, short lecture, pamphlet, and giving direction and guidance. This intervention was investigated and finalized after a basic study. In the second stage (posttest) and after 45 days of intervention, the participants filled out the questionnaires once again.

It is noteworthy to mention that gathering data was done after gaining a letter of introduction and getting permission. Upon visiting,, researcher introduces herself, expressed the aims of the research and observed all ethical considerations. The researcher interviewed illiterate women and gathered their data with no manipulation. Furthermore, all ethical considerations including anonymous questionnaires, justification of nature and aims of research, and voluntarily participation were observed in this research.

### 3.4. Statistical Analysis

The normality of data was investigated with Kolmogorov-Smirnov test Then, descriptive statistics such as frequency distribution, mean, and standard deviation and analytical statistics such as chi-square statistical test, Pearson correlation coefficient, independent samples t-test, one-way ANOVA, and paired t-test were used. Data were analyzed with SPSS software (version 20) and the significance level was considered to be 0.05.

## 4. Results

Due to having exclusion criteria, seven individuals left this research and data were analyzed for 84 individuals. Mean age of the women was 12.1 ± 37.4 years ranging from 16 to 71. The average of the number of children among participants was 2.1 ± 3.7 with minimum of one child and maximum of 9 children. Most of the women participating in the study were illiterate (35%), housewife (91.2%), having both sons and daughters (70.3%), from village (56%) and owned their houses (68.1%) (Table 1). Thirty-eight individuals (41.8%) replied that they were living with the families of their husbands. Regarding the employment status of their husbands, 56% of their husbands (51 individuals) were workers, 26.4% (24 individuals) were self-employed, and 17.6% of them (16 persons) were jobless. In addition, 35.2% (32 individuals) and 14.3% (13 individuals) of participants reported that their husbands smoked and were addicted to drugs, respectively. The results of chi-square test showed that the frequency of domestic violence against women participants were significantly different before and after the educational intervention (Table 2). In other words, it seems that the prevalence of domestic violence against women participants decreased after educational intervention.

The results of the study using paired t-test indicated that average scores for total awareness, attitude, self-esteem, self-efficacy, and empowerment constructs were significantly different after educational intervention, as compared to pre-intervention period. In other words, it seems that educational program based on empowerment model has been effective in improving the average scores of aforementioned constructs and total empowerment (Table 3). In bivariate analysis, the results of Pearson correlation test, independent t test, and one-way ANOVA showed there are statistically significant relations between awareness construct and the variable of living with husband's family (P = 0.003) and attitude construct and variable of level of education (P = 0.01) and variable of living with husband's family (P = 0.02). In addition, there are statistically significant relations between self-esteem construct and variables of age (P = 0.04), length of married life (P = 0.03), number of children (P = 0.04), level of education (P = 0.02), and living with husband's family (P = 0.01). Eventually, there were significant relations between total power variable with variables of level of level of education (P = 0.03), employment status (housewife-employed) (P = 0.02), and living with husband's family (P = 0.002). No statistically significant relations were found between demographic variables and self-efficacy construct (P < 0.05).

**Figure 1.**
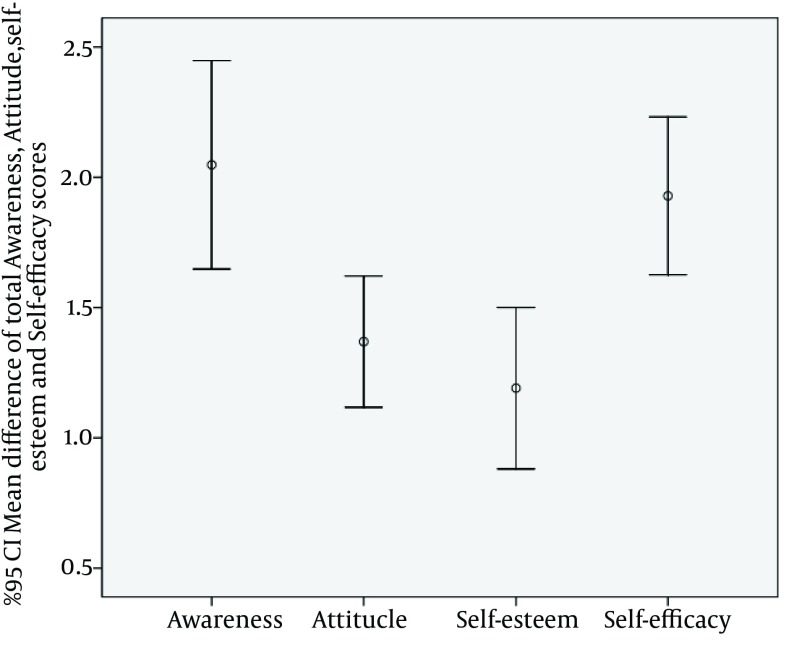
Comparing the Mean Difference of total Awareness, Attitude, Self-esteem and Self-efficacy scores in Before and after intervention

We compared the mean difference total awareness, attitude, self-esteem and self-efficacy scores. The mean difference of total awareness, attitude, self-esteem and self-efficacy scores calculated by mean total awareness, attitude, self-esteem and self-efficacy scores in pre intervention minus mean total awareness, attitude, self-esteem and self-efficacy scores in post intervention. It was significantly greater in post intervention than the in pre intervention (P < 0.05). These results were presented by error bar plot in Figure 1.

**Table 1.** Characteristics of Participants (N = 84)

Characteristics of Participants	No. (%)
**Age, y**	
Less 25	16 (17.9)
25-35	29 (24.2)
35-45	22 (31.9)
Over 45 years	24 (26.4)
**Education level**	
Illiterate	32 (35)
Elementary education	27 (29.7)
Middle school graduate	19 (20.9)
High school graduate	13 (14.4)
**Job**	
Housekeeper	83 (91.2)
Employee	8 (8.8)
**Location**	
Urban	40 (44)
Rural	51 (56)
**Gender of children**	
Only girl	16 (17.6)
Boy and girl	75 (82.4)

**Table 2.** Distribution of Violence Before and After the Educational Intervention^a^

	Before Education	After Education
**Has violence**	41 (45.1)	12 (13.2)
**No violence**	50 (54.9)	72 (79.1)
**Total**	91 (100)	84 (92.3)

^a^ P value = 0.001, P ˂ 0.0, 0.5 sig.

**Table 3.** The Mean and Standard Deviation of the Distribution of Structural Empowerment Scores Before and After the Intervention in Participants^a^

	After Intervention	Before Intervention
**Knowledge**	6.53 ± 2.44	8.54 ± 2
**Attitude**	4.14 ± 2.17	5.53 ± 2.13
**Self-efficacy**	5.29 ± 1.92	6.64 ± 1.39
**Self-esteem**	5.24 ± 1.66	7.19 ± 1.29
**Total empowerment**	21.55 ± 5.44	28.05 ± 4.77

^a^ P Value < 0.001.

## 5. Discussion

The results of the current study showed that there was a significant decrease in violence against women after intervention, as compared to pre-intervention period. This indicates that implementing women empowerment program has significantly decreased domestic violence against women which confirms the impact of women empowerment program in decreasing violence against women on preventing violence. The results of Kim et al. study (16) showed that educational intervention and educational pamphlets about domestic violence against women decrease husbands' violence against women. Also Lako et al. study (7) revealed that educational interventions for the homeless decrease violence among them. The study of Noughani et al. (17), however, showed that education has no impact on decreasing violence. Therefore, this research suggests that violence against women can be decreased by educating women, detecting ways to curb anger, making women aware of communication skills, and increasing self-confidence.

The results of the study using paired t-test showed that that average scores for awareness, attitude, self-esteem, self-efficacy, and total power constructs were significantly different after educational intervention, as compared to pre-intervention period. In other words, it seems that educational program based on empowerment model has been effective in improving the average scores of aforementioned constructs and total empowerment. Ramsay et al. study (6) showed that one of the reasons leading to the use of violence by husbands against women is women low awareness of violence and lack of awareness of the other side's rights. Soleiman Ekhtiari et al. showed that society-centered education increases the awareness about violence and individual's rights and results in the decrease of violence against women (1). These results were in line with those of this study. Thus, to improve women awareness of their rights and increase life skills, this research suggests providing education via mass media. Furthermore, it is necessary to hold educational classes in neighborhoods and communities of homemakers such as mosques. Boroumandfar et al. showed there has been a statistical significant change in the attitude toward violence after intervention, as compared to preintervention period (18). Ramsay et al. study conducted on health-care personnel about violence showed that participating physicians had a higher attitude toward violence as compared to other treating staff (6).

Investigating the impact of educational intervention showed that participants' self-efficacy changed significantly after the study, which indicated educational intervention is an important indicator in individuals' self-efficacy. Participating in educational classes and interacting with others change women from passive to active individuals, involved in social dealings. Expressing one's experiences and using vicarious experiences, getting exposed to successful experiences through setting accessible goals and increasing the possibility of having successful operation, using verbal awards, education and learning useful lessons from failure as one does from success, and lack of ownership feeling toward others increase one's self-efficacy. It seems that one reason for significant difference in self-efficacy score is the involvement of the individual in education by having a role, expressing her experiences, being permitted to express her ideas in sessions and effective encouragements in the classroom.

The results of the study showed that there was a significant difference in women self-esteem scores toward violence after intervention. The significant change in self-esteem scores before and after intervention indicated that education had a significant impact on improving individuals' self-esteem in this study. Moshki et al. showed that educating life skills to individuals increases their skills (problem-solving skill, saying no skill, self-expression, curbing anger, etc.) and empowers the individual to actualize their knowledge, attitude, and values and thus have healthy motivation and behavior (19).

The results of the current study showed that there was a significant difference in the score of women power toward violence. This significant difference in the power score before and after education (P < 0.001) confirmed the impact of empowerment program in increasing women general power in preventing violence. The results of the current research were in line with those of Soleiman Ekhtiari et al., which showed an increase in power score after education (1).

### 5.1. Strengths, Limitations and Suggestions

Lack of any control groups is a limitation of the present study and educating participants with modern educational methods is among the strengths of this research. Utilizing empowerment strategies such as creating self-help groups, implementing educational group programs, and gaining other people's support is a part of empowerment programs are among other strengths. As one of the manifestations and the moving force of empowerment, education is the first major strategy in codifying, designing, and implementing empowerment programs. In these programs, education is done in groups, so that they can exchange ideas, interact with each other, and use each other's support. All these can increase women power in maintaining and improving their psychological health.

Difficulties in supplying and equipping educational accessories, possible changes in the time of some educational sessions, and lack of similar articles to compare empowerment constructs and demographic variables are among the restrictions of this research.

Educating life skills to women lead to gaining experience, solving problems, having effective communications, and behaving with courage, all of which prevent showing negative behaviors at home by women. This increases their self-confidence and prevents their husbands to use violent behavior against them. Educating life skills to improve self-esteem, curb anger, and prevent domestic violence is founded on the hypothesis that people with low self-esteem easily put up with their spouse's violence and this is a deficiency in people's interpersonal and intrapersonal functions, which includes being incompetent in problem-solving, decision- making , critical thinking, and refusing. Therefore, the purpose of educating life skills is to educate and increase interpersonal and intrapersonal functions to manage and solve life problems.
